# Blood Tells

**Published:** 1871-10

**Authors:** 


### BLOOD TELLS.

In former years animal and human blood seemed to be too near alike for discrimination, and yet the life of a human being hangs upon the distinction; hence great attention has been paid to the subject by recent researches. Blood is composed of little living bodies, round and flat, called blood-disks; these measure largest in animals that live in air and water, such as frogs, salamanders, etc.; fishes and birds come next; then man, with the other animals which are first fed with the mother's milk; but as the size of man's disks depended on the state of the health, the difference was not sufficiently great to authorize the determination certainly whether a particular specimen was the blood of an animal or man. But more minute investigations show that if a drop of blood at the temperature of sixty degrees is watched with a microscope, it slowly breaks up into a net-work; this net-work is different in different animals and at different times. A man's blood forms quickly into a small pattern, that of animals into larger patterns, and more slowly. Each kind of blood has its own pattern, and no doubt the time will come when it may be determined with absolute accuracy whether any particular specimen of blood is from a man, a monkey, or an elephant.

